# Predictors of hospitalization of tuberculosis patients in Montreal, Canada: a retrospective cohort study

**DOI:** 10.1186/s12879-016-1997-x

**Published:** 2016-11-15

**Authors:** Lisa A. Ronald, J. Mark FitzGerald, Andrea Benedetti, Jean-François Boivin, Kevin Schwartzman, Gillian Bartlett-Esquilant, Dick Menzies

**Affiliations:** 1 Department of Epidemiology, Biostatistics and Occupational Health, McGill University, Montreal, QC Canada; 2 Division of Respiratory Medicine, Faculty of Medicine, University of British Columbia, Vancouver, BC Canada; 3 Centre for Clinical Epidemiology and Evaluation, Vancouver Coastal Health Research Institute, Vancouver, BC Canada; 4 Institute for Heart and Lung Health, University of British Columbia, Vancouver, Canada; 5 Respiratory Epidemiology and Clinical Research Unit (RECRU)/ Montreal Chest Institute, McGill University Health Centre, Room 419, 2155 Guy St, Montreal, QC H3H 2R9 Canada; 6 Department of Family Medicine, McGill University, Montreal, QC Canada; 7 Department of Medicine, McGill University, Montreal, QC Canada

**Keywords:** Active tuberculosis, Health service utilization, Length of hospital stay, Survival analysis, Epidemiology

## Abstract

**Background:**

Hospitalization is the most costly health system component of tuberculosis (TB) control programs. Our objectives were to identify how frequently patients are hospitalized, and the factors associated with hospitalizations and length-of-stay (LOS) of TB patients in a large Canadian city.

**Methods:**

We extracted data from the Montreal TB Resource database, a retrospective cohort of all active TB cases reported to the Montreal Public Health Department between January 1996 and May 2007. Data included patient demographics, clinical characteristics, and dates of treatment and hospitalization. Predictors of hospitalization and LOS were estimated using logistic regression and Cox proportional hazards regression, respectively.

**Results:**

There were 1852 active TB patients. Of these, 51% were hospitalized initially during the period of diagnosis and/or treatment initiation (median LOS 17.5 days), and 9.0% hospitalized later during treatment (median LOS 13 days). In adjusted models, patients were more likely to be hospitalized initially if they were children, had co-morbidities, smear-positive symptomatic pulmonary TB, cavitary or miliary TB, and multi- or poly-TB drug resistance. Factors predictive of longer initial LOS included having HIV, renal disease, symptomatic pulmonary smear-positive TB, multi- or poly-TB drug resistance, and being in a teaching hospital.

**Conclusions:**

We found a high hospitalization rate during diagnosis and treatment of patients with TB. Diagnostic delay due to low index of suspicion may result in patients presenting with more severe disease at the time of diagnosis. Earlier identification and treatment, through interventions to increase TB awareness and more targeted prevention programs, might reduce costly TB-related hospital use.

## Background

Tuberculosis (TB) is generally considered an ambulatory disease [1], however, hospitalization remains an important part of management [2, 3]. Benefits of hospitalization include isolation of infectious patients, and medical stabilization of patients with or at-risk of complications of the disease or therapy [1]. For patients who are homeless or living in group settings, have psychiatric or substance abuse issues, or have difficulties with activities of daily living, extended inpatient treatment may be used to ensure completion of treatment [4, 5].

However, hospitalization increases risk of nosocomial transmission to health care workers and other vulnerable patients [6]. Inpatient diagnostic delays can increase transmission risk in this vulnerable population [7, 8]. TB hospitalizations can lead to psychological difficulties for patients due to isolation and detention [1]. Lastly, there are cost implications [2, 9, 10]—hospitalizations are generally the largest health system cost component of TB case management, often accounting for more than 50% of total treatment costs [11–15].

Better understanding of which patients are hospitalized during the diagnostic and management phases of TB can help policy-makers and clinicians more efficiently plan treatment programs and resource needs. Therefore, the objectives of the current study were to identify how frequently patients were hospitalized, and what factors were associated with hospitalization and length of hospital stay during diagnosis and treatment of active TB patients in a large Canadian city, using patient-level clinical and demographic data from a clinical research database.

## Methods

### Data source and study population

A retrospective cohort was identified of all individuals with confirmed TB, and notified to Montreal Public Health between January 1996 and May 2007. Given the mandatory legal requirement to report all TB cases, this is virtually a complete capture of all persons diagnosed with microbiologically or clinically confirmed TB in Montreal (covering a catchment of approximately two million people). Further, approximately two-thirds of TB cases in Quebec are reported in Montreal [16], thus the database includes the majority of TB cases in the province for those years. Detailed information regarding patients’ clinical status, treatment, and hospitalization history for Montreal Island hospitals was extracted from public health charts and from medical charts at treating hospitals and clinics for each case. Data extraction was performed by trained research assistants using standardized data extraction forms. Ethical approval for this study was obtained through the McGill University Health Centre Research Ethics Board.

### Outcome and predictor variables

The main outcomes were whether or not the individual had a TB-related hospitalization and the length of stay (LOS) for the hospitalizations. Hospitalizations were defined as admissions to an acute care hospital on the Island of Montreal, and excluded emergency department visits of less than 24 h, hospital day clinics, and day surgeries. Hospital transfers were defined as when a discharge and a new admission date were on the same or next day. When a transfer was identified, these were counted as one continuous hospitalization. LOS was calculated for each continuous hospitalization as the difference between date of discharge (or in-hospital death) and admission date. We excluded hospitalizations missing admission or discharge dates (*n* = 19).

The TB diagnosis date was identified as the treatment start date, or when not available, the date the mandatory notification was made. Hospitalizations were stratified based on whether the timing was at the time of diagnosis or during treatment—defined as: 1) an initial TB-related hospitalization (admission within 1 month before or up to 1 month after the date of TB diagnosis, or where TB was diagnosed in-hospital); and 2) a hospitalization later during the treatment period (admission more than 1 month after the TB diagnosis date and while the patient was still undergoing TB treatment) [14]. It was hypothesized that factors associated with hospitalizations at the time of diagnosis and treatment initiation would differ from those associated with hospitalizations occurring later during treatment.

Demographic variables included age in years, sex, provincial health insurance registration, self-reported Aboriginal status, immigration details, and homeless status. We stratified foreign-born patients into categories of immigration from high or low-moderate TB incidence countries based on WHO definitions [17], and time since immigration based on published studies indicating that TB rates are highest within the first 2 years of arrival [18]. Additional variables included smoking, drug and alcohol abuse history, HIV co-infection, and clinical characteristics (smear and culture results, disease site, TB drug resistance, cavitary and miliary TB, pulmonary and/or systemic symptoms), and method of therapy supervision (directly observed therapy (DOT) or self-administered therapy (SAT)). Patient co-morbidities were extracted from notations in hospital and public health charts; these included diabetes, cancer, renal disease, HIV, substance abuse, liver disease or other conditions. Among hospitalized patients, we identified if the hospital was a teaching (university) or non-teaching hospital, and if they died while in-hospital.

### Statistical analyses

We calculated the proportion of patients with TB-related hospitalizations, overall, and stratified by hospitalization timing, and the mean (standard deviation) and median (interquartile range, IQR) LOS (in days). We further estimated the proportion of patients starting treatment in-hospital, and for these patients, estimated the length of in-hospital diagnostic delay as the median (IQR) number of days between date of hospital admission and date when TB treatment was started.

We identified factors associated with having any hospitalization by calculating univariable and multivariable odds ratios using logistic regression (SAS 9.3, SAS Institute Inc, Cary, USA), stratified on hospitalization timing. We considered variables for inclusion in adjusted models using a backward selection procedure.

We identified factors predictive of hospital LOS using Cox proportional hazards regression [19], whereby survival time was duration of time spent in-hospital and the event was hospital discharge. Patients were censored when they died in-hospital. We included hospital type in the model as a time-dependent covariate to account for transfers. Since some patients had multiple separate hospitalizations, we used a conditional counting process model whereby each hospitalization was assigned to a separate stratum and the time scale was time since study entry [20]. This allowed the baseline hazard to differ depending on whether it was the patient’s first or second hospitalization [20]. We used a robust sandwich variance estimator for estimating standard errors. When there was strong evidence that proportional hazards assumptions did not hold, we included an interaction term in the model of the variable multiplied by time. We considered variables for inclusion in adjusted models using a backward selection procedure.

## Results

### Characteristics of TB patients

There were 1852 patients with confirmed TB reported to Montreal Public Health between January 1996 and May 2007 (Table 1). Roughly half were male, with a median age of 44 years, and 80.5% were foreign-born, with slightly more than one-quarter having arrived to Canada within 2 years before the TB notification date. Approximately one-quarter were current or past smokers, almost one-sixth reported past or current substance abuse, but few were homeless. Six patients were reported as Canadian-born Aboriginal (data not shown). Concomitant HIV infection was documented in 7.9% of patients (Table 1).

**Table 1:** Characteristics of persons with TB, notified to Montreal public health between January 1996 -- May 2007, *N* = 1852

	Number (%) of active TB patients^a^	
	N	%
*Patient demographics*		
Sex- male	992	53.6
Age group		
0–19 years	143	7.7
20–34 years	602	32.5
35–64 years	711	38.4
65+ years	390	21.1
Had provincial health insurance coverage	1499	80.9
Country of birth^b^		
Canada	317	17.1
Foreign-born, low/moderate TB incidence country	1112	60.0
Foreign-born, high TB incidence country	380	20.5
Immigration, years since arrival		
≤ 2 years	485	26.2
3–14 years	541	29.2
15 or more years	338	18.3
Foreign-born, unknown year of arrival	128	6.9
Canadian-born	317	17.1
Homeless	9	0.5
*Patient co-morbidities*		
Cancer	41	2.2
Diabetes	132	7.1
HIV positive	146	7.9
Renal disease	105	5.7
Liver disease	153	8.3
Ever substance abuse	249	13.4
Number of co-morbidities^c^		
2 or more	412	22.3
1	446	24.1
Ever smoker	489	26.4

^a^Missing data: sex (*n* = 19), age (*n* = 6), country of birth (*n* = 43)

^b^High TB incidence countries (WHO estimated sputum smear positive TB rate of ≥15 per 100,000, 3 year average, 2005–2007) [28]

^c^Co-morbidities include renal disease, liver disease, diabetes, cancer, substance abuse, HIV infection, or other

Most patients had pulmonary TB disease, of which one-third were smear-positive (Table 2). Few strains were TB drug resistant (6.6% mono-resistant and 1.8% multi/poly-drug resistant). Most patients were cured (82%), while 6.9% died and the remainder moved out of province, defaulted or failed treatment (Table 2). The median treatment duration was 208 days (IQR = 183–295) and was longest for multi- and poly-drug resistant cases (median 517 days, data not shown). Further stratification of covariates by years since immigration suggested that recent immigrants (within the past 2 years) were on average younger and had less co-morbidity than Canadian-born active TB patients and foreign-born patients in Canada for 15 or more years, were more likely to have pulmonary TB that was smear-negative and less likely to be symptomatic at the time of presentation (Table 3).

**Table 2:** Clinical characteristics of persons with TB notified to Montreal Public Health between January 1996 -- May 2007, *N* = 1852

	Number (%) of active TB patients^a^	
	N	%
Mycobacteriology and disease site		
Pulmonary, Smear+	619	33.4
Pulmonary, Smear-	625	33.7
Extra-pulmonary	563	30.4
Clinical and radiographic features of TB disease		
Miliary TB	35	1.9
Cavitary TB	336	18.1
Any pulmonary TB-related symptoms^b^	1530	82.6
Any systemic TB-related symptoms^c^	1018	55.0
Drug resistance		
Multi- and poly-TB drug resistant	33	1.8
Mono-TB drug resistant	122	6.6
Pan-sensitive	1664	89.8
Therapy supervision, ever directly-observed (DOT)	807	43.5
Treatment duration in days, median (min-max)	208 (183–295)	
Treatment outcome		
Cured	1518	82.0
Died	127	6.9
Defaulted/failed	34	1.8
Moved	69	3.7

^a^Missing data: TB diagnosis (*n* = 45), drug resistance (*n* = 33), treatment outcome (*n* = 104)

^b^Pulmonary TB-related symptoms: cough, hemoptysis, and abnormal chest x-ray

^c^Systemic TB-related symptoms: weight loss, fever, fatigue, night sweats

**Table 3:** Characteristics and treatment outcomes of persons notified to Montreal public health between January 1996 -- May 2007, stratified by years since arrival to Canada

Characteristics of Active TB Patients^a^	Foreign-born, years since arrival to Canada						Canadian-born		*p*-value
	0–2 (*N* = 485)		3–14 (*N* = 541)		15+ (*N* = 338)		(*N* = 317)		
	*n*	%	*N*	%	*N*	%	*n*	%	
Sex, male	281	(57.9)	275	(50.8)	172	(51.0)	172	(54.2)	0.22
Age group, in years									<0.01
0–19	51	(10.5)	35	(6.5)	1	(0.3)	50	(15.8)	
20–34	257	(53.0)	233	(43.0)	40	(11.8)	37	(11.7)	
35–64	147	(30.3)	222	(41.0)	169	(50.0)	105	(33.1)	
65+	29	(6.0)	50	(9.2)	128	(37.9)	122	(38.5)	
Had provincial health insurance #	213	(43.9)	499	(92.2)	328	(97.0)	308	(97.2)	
Homeless	1	(0.2)	0	(0)	1	(0.3)	7	(2.2)	<0.01
Ever smoker	122	25.2	99	18.3	98	29.0	159	50.2	<0.01
Ever substance abuse	27	(5.6)	56	(10.4)	51	(15.1)	98	(30.9)	<0.01
HIV	35	(7.2)	51	(9.4)	21	(6.2)	28	(8.8)	0.38
Number of co-morbidities^b^									<0.01
2 or more	53	(10.9)	78	(14.4)	104	(30.8)	130	(41.0)	
1	93	(19.2)	116	(21.4)	102	(30.2)	92	(29.0)	
TB diagnosis									<0.01
Pulmonary, Smear+	126	(26.0)	188	(34.8)	123	(36.4)	144	(45.4)	
Pulmonary, other	257	(53.0)	124	(22.9)	77	(22.8)	108	(34.0)	
Extra-pulmonary	88	(18.1)	219	(40.5)	131	(38.8)	55	(17.4)	
Any pulmonary symptoms^c^	435	(89.7)	403	(74.5)	273	(80.8)	291	(91.8)	<0.01
Any systemic symptoms^d^	201	(41.4)	321	(59.3)	212	(62.7)	203	(64.0)	<0.01
Multi- or poly-drug resistant	13	(2.7)	12	(2.2)	2	(0.6)	4	(1.3)	0.20
Treatment outcome									<0.01
Cured	421	(86.8)	465	(86.0)	263	(77.8)	246	(77.6)	
Died	3	(0.6)	16	(3.0)	41	(12.1)	45	(14.2)	
Defaulted/failed	7	(1.5)	10	(1.8)	4	(1.2)	9	(2.8)	
Moved	34	(7.0)	20	(3.7)	5	(1.5)	3	(1.0)	

^a^Note: table subset to people with known birth country and timing of arrival to Canada (*N* = 1681). Missing data: sex (*n* = 18), age (*n* = 5), TB diagnosis (*n* = 41), treatment outcome (*n* = 89)

^b^Co-morbidities include renal disease, liver disease, diabetes, cancer, substance abuse, HIV infection, or other (as reported in public health or hospital records)

^c^Pulmonary TB-related symptoms include cough, hemoptysis, and/or abnormal chest x-ray

^d^Systemic TB-related symptoms include weight loss, fever, fatigue, and/or night sweats

### Predictors of hospitalizations and LOS

**Fig. 1:**
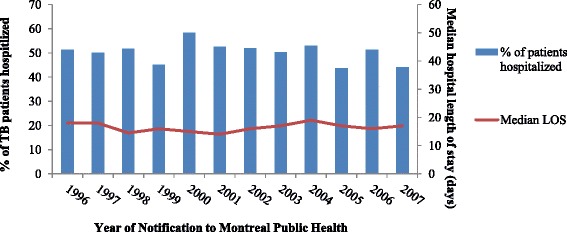
Number of initial hospitalizations and median length of hospital stay, notified to Montreal Public Health between January 1996 -- May 2007, stratified by year of notification (*N* = 1852)

In total, 1001 patients were hospitalized (54.1%); 942 patients (50.9%) had a hospitalization initially, and 167 (9.0%) later during treatment (Table 4). Of 942 hospitalized initially, 114 were hospitalized more than once during this period 30 days before or after the TB diagnosis date (Table 4). The median LOS of initial hospitalizations was 17.5 days (IQR = 9–31) and 13 days (IQR = 6–22) later during treatment. Treatment was started in-hospital for 731 patients (39.5%), with a median time to starting treatment after admission of 4 days (IQR = 2–8). The proportion of patients hospitalized and median LOS did not change over the study period (Fig. 1).

**Table 4:** Number of hospitalizations and lengths of hospital stay of active TB cases, notified to Montreal public health between January 1996 -- May 2007, stratified by timing of hospitalization

	Timing of hospitalization		All hospitalizations
	Initial^a^	During^b^	
Patients with any hospitalization, *n* (%)	942 (50.9)	167 (9.0)	1001 (54.1)
Patients with >1 hospitalization, *n* (%)	114 (6.1)	35 (1.9)	124 (6.7)
Total number of hospitalizations	1006	206	1212
Mean length of hospital stay (LOS), in days (se)	28.2 (39.0)	21.6 (36.1)	27.2 (38.6)
Median length of hospital stay (LOS), in days (IQR)	17.5 (9–31)	13 (6–22)	16.5 (8–29)
Patients starting TB treatment in-hospital, *n* (%)	731 (39.5%)	-	731 (39.5%)
Median # days (IQR) from admission to treat start	4 (2–8)	-	4 (2–8)

*LOS* length of stay, *se* standard error, *IQR* interquartile range

^a^Initial = hospital admission within 1 month before start of TB treatment or up to 1 month after treatment start, or any admission where TB treatment started in-hospital

^b^During = hospital admission more than 1 month after start of TB treatment and during TB treatment period

In adjusted models (Table 5), patients were more likely to be hospitalized initially if they were children, had HIV co-infection, renal disease, co-morbidities, smear-positive pulmonary TB, cavitary TB, miliary TB, pulmonary or systemic symptoms, and multi- or poly-TB drug resistance. Factors associated with hospitalization later during treatment were similar to those associated with initial hospitalizations (Table 5).

**Table 5:** Predictors of hospitalization during diagnosis and treatment of active TB cases, notified to Montreal public health between January 1996 -- May 2007, stratified by timing of hospitalization (*N* = 1852)

Characteristics	Any initial hospitalization (yes/no)			Any hospitalization during treatment (yes/no)		
	Number of patients (%)	Crude OR (95% CI)	Adjusted OR^a^ (95% CI)	Number of patients (%)	Crude OR (95% CI)	Adjusted OR^a^ (95% CI)
Male	536 (54.0)	1.30 (1.07–1.57)	-	91 (9.2)	1.08 (0.78–1.49)	-
Female	399 (47.4)	1.00		72 (8.6)	1.00	
Age group						
0–19 years	85 (59.4)	2.04 (1.38–3.00)	2.52 (1.63–3.92)	15 (10.5)	2.09 (1.10–3.97)	2.82 (1.38–5.76)
20–34 years	279 (46.4)	1.00	1.00	32 (5.3)	1.00	1.00
35–64 years	249 (54.7)	1.39 (1.11–1.73)	1.09 (0.83–1.43)	60 (8.7)	1.64 (1.05–2.56)	1.11 (0.68–1.81)
≥ 65 years	258 (66.2)	1.92 (1.47–2.51)	1.13 (0.80–1.59)	58 (14.9)	3.11 (1.98–4.89)	2.19 (1.25–3.85)
Had provincial health insurance #						
Yes	804 (53.6)	1.75 (1.37–2.24)	-	149 (9.9)	2.05 (1.24–3.40)	-
No	138 (39.1)	1.00		18 (5.1)	1.00	
Country of birth			-			-
Canada	196 (61.8)	2.02 (1.48–2.75)		51 (16.1)	2.61 (1.59–4.30)	
Foreign, low-moderate TB incidence	551 (49.6)	1.16 (0.92–1.47)		85 (7.6)	1.13 (0.72–1.78)	
Foreign, high TB incidence country	174 (45.8)	1.00		26 (6.8)	1.00	
Immigration, years since arrival						
Foreign-born, unknown arrival date	53 (41.4)	0.41 (0.27–0.63)	-	13 (10.2)	0.59 (0.31–1.13)	-
15 or more years	204 (60.4)	0.90 (0.65–1.25)		37 (11.0)	0.64 (0.41–1.01)	
3–14 years	269 (49.7)	0.58 (0.43–0.77)		39 (7.2)	0.41 (0.26–0.63)	
0–2 years	199 (41.0)	0.41 (0.30–0.54)		22 (4.5)	0.25 (0.15–0.42)	
Canadian-born	196 (61.8)	1.00		51 (16.1)	1.00	
Homeless			-			-
Yes	8 (88.9)	7.46 (0.93–59.66)		2 (22.2)	2.91 (0.60–14.11)	
No	934 (50.7)	1.00		165 (9.0)	1.00	
Cancer			-			
Yes	30 (73.2)	2.23 (1.09–4.53)		13 (31.7)	5.00 (2.54–9.84)	3.43 (1.63–7.23)
No	912 (50.4)	1.00		154 (8.5)	1.00	1.00
Diabetes			-			-
Yes	98 (74.2)	2.83 (1.88–4.26)		21 (15.9)	2.04 (1.24–3.35)	
No	844 (49.1)	1.00		146 (8.5)	1.00	
HIV						
Positive	111 (76.0)	3.24 (2.17–4.85)	1.46 (0.90–2.36)	36 (24.7)	3.84 (2.60–5.97)	3.31 (1.96–5.60)
Negative or unknown	831 (48.7)	1.00	1.00	131 (7.7)	1.00	1.00
Renal disease						-
Yes	83 (79.1)	3.92 (2.38–6.47)	1.99 (1.10–3.59)	22 (21.0)	2.93 (1.78–4.83)	
No	859 (49.2)	1.00	1.00	145 (8.3)	1.00	
Liver disease						-
Yes	105 (68.6)	2.02 (1.40–2.92)	-	25 (16.3)	2.14 (1.35–3.40)	
No	837 (49.3)	1.00		142 (8.4)	1.00	
Ever substance abuse						-
Yes	176 (70.7)	2.69 (1.99–3.63)	-	32 (12.9)	1.60 (1.06–2.42)	
No	766 (47.8)	1.00		135 (8.4)	1.00	
Number of co-morbidities						
2 or more	316 (77.3)	5.31 (4.04–6.98)	3.56 (2.50–5.07)	76 (18.6)	5.44 (3.64–8.14)	2.90 (1.69–4.96)
1	242 (53.9)	1.84 (1.46–2.32)	1.43 (1.08–1.89)	51 (11.4)	3.06 (1.99–4.70)	2.17 (1.31–3.60)
0	384 (38.6)	1.00	1.00	40 (4.0)	1.00	1.00
Ever smoker						-
Yes	306 (62.6)	1.92 (1.54–2.39)	-	61 (12.5)	1.69 (1.21–2.36)	
No	636 (46.7)	1.00		106 (7.8)	1.00	
TB diagnosis						-
Pulmonary, Smear+	447 (72.2)	3.87 (3.02–4.96)	1.48 (1.04–2.09)	70 (11.3)	1.43 (0.97–2.12)	
Pulmonary, Smear-	257 (41.2)	1.05 (0.83–1.32)	0.65 (0.46–0.90)	47 (7.5)	0.91 (0.60–1.40)	
Extra-pulmonary	228 (40.5)	1.00	1.00	46 (8.2)	1.00	
Cavitary TB						-
Yes	230 (68.5)	2.49 (1.92–3.23)	1.75 (1.28–2.40)	25 (7.4)	0.76 (0.48–1.19)	
No	712 (47.0)	1.00	1.00	142 (9.4)	1.00	
Miliary TB						
Yes	29 (82.9)	5.33 (2.05–13.87)	3.53 (1.26–9.88)	8 (22.9)	3.09 (1.38–6.92)	2.09 (0.88–4.96)
No	913 (50.3)	1.00	1.00	159 (8.8)	1.00	1.00
Any pulmonary TB-related symptoms						
Yes	853 (55.8)	3.45 (2.63–4.53)	2.12 (1.44–3.11)	149 (9.7)	1.77 (1.07–2.93)	-
No	89 (27.6)	1.00	1.00	18 (5.6)	1.00	
Any systemic TB-related symptoms						
Yes	697 (68.5)	5.18 (4.22–6.36)	3.54 (2.82–4.46)	123 (12.1)	2.44 (1.70–3.50)	1.72 (1.16–2.57)
No	245 (28.4)	1.00	1.00	44 (5.3)	1.00	1.00
Multidrug or poly-drug resistance						
Yes	26 (78.8)	3.95 (1.61–9.67)	6.98 (2.65–18.30)	8 (24.2)	3.48 (1.54–7.89)	5.55 (2.18–14.14)
No	914 (51.2)	1.00	1.00	157 (8.8)	1.00	1.00

*OR* odds ratio, *CI* confidence interval

^a^Models adjusted for all reported variables

Factors predictive of longer initial LOS in adjusted models included older age, HIV infection, renal disease, pulmonary smear-positive TB, pulmonary symptoms, multi- or poly-TB drug resistance, and admission to a teaching hospital (Table 6). Factors associated with longer LOS when hospitalized later during treatment were similar to initial hospitalizations, with the exception that having extra-pulmonary TB was associated with longer LOS (Table 6).

**Table 6:** Predictors of hospital length of stay during diagnosis and treatment of active TB, stratified by timing of hospitalization, Montreal, January 1996 -- May 2007

	Initial hospitalizations (*n* = 1006)		Hospitalizations during treatment (*n* = 206)	
Characteristics	Crude HR (95% CI)	Adjusted HR^a,b^ (95% CI)	Crude HR (95% CI)	Adjusted HR^a^ (95% CI)
Sex				
Male	1.01 (0.89–1.14)	-	1.29 (0.78–2.11)	-
Female	1.00		1.00	
Age in years^c^				0.99 (0.98–1.00)
log(LOS) ≤ 2.5	1.02 (1.02–1.03)	1.02 (1.02–1.03)	-	-
log(LOS) > 2.5	0.96 (0.96–0.96)	0.96 (0.96–0.96)	-	-
Had provincial health insurance #				
Yes	0.96 (0.81–1.14)	-	1.15 (0.63–2.10)	-
No	1.00		1.00	
Country of birth				
Canada	0.68 (0.55–0.83)	-	0.80 (0.47–1.37)	-
Foreign, low-moderate incidence	0.88 (0.74–1.04)		0.57 (0.33–0.98)	
Foreign, high incidence country	1.00		1.00	
Immigration, years since arrival				
Foreign-born, unknown arrival date	1.47 (1.13–1.91)	-	0.57 (0.28–1.15)	-
15 or more years	1.11 (0.92–1.35)		0.70 (0.34–1.44)	
3–14 years	1.50 (1.26–1.79)		0.85 (0.50–1.42)	
0–2 years	1.42 (1.17–1.71)		1.15 (0.58–2.28)	
Canadian-born	1.00		1.00	
Homeless		-		-
Yes	0.89 (0.56–1.40)		0.26 (0.11–0.63)	
No	1.00		1.00	
Cancer		-		
Yes	0.82 (0.55–1.23)		1.42 (0.89–2.26)	-
No	1.00		1.00	
Diabetes		-		
Yes	0.92 (0.76–1.11)		1.05 (0.63–1.75)	-
No	1.00		1.00	
HIV				
Positive	0.83 (0.70–0.99)	0.77 (0.66–0.90)	0.93 (0.59–1.47)	0.61 (0.40–0.91)
Negative or unknown	1.00	1.00	1.00	1.00
Renal disease				
Yes	0.55 (0.43–0.70)	0.59 (0.46–0.75)	0.45 (0.21–0.95)	0.58 (0.31–1.10)
No	1.00	1.00	1.00	1.00
Liver disease				
Yes	0.79 (0.66–0.94)	-	1.62 (0.99–2.66)	-
No	1.00		1.00	
Ever substance abuse				
Yes	0.91 (0.79–1.05)	-	0.88 (0.58–1.33)	-
No	1.00		1.00	
Number of co-morbidities				
2 or more	0.65 (0.56–0.75)	-	0.74 (0.44–1.25)	-
1	0.85 (0.73–1.00)		0.69 (0.36–1.31)	
0	1.00		1.00	
Ever smoker				
Yes	0.84 (0.74–0.96)	-	0.91 (0.59–1.40)	-
No	1.00		1.00	
TB diagnosis				
Pulmonary, Smear+	0.61 (0.51–0.72)	0.64 (0.54–0.77)	1.51 (0.84–2.69)	1.26 (0.78–1.99)
Pulmonary, Smear-	089 (0.73–1.09)	1.02 (0.84–1.26)	1.50 (0.80–2.83)	1.81(1.07–3.04)
Extra-pulmonary	1.00	1.00	1.00	1.00
Cavitary TB				
Yes	0.92 (0.90–1.05)	-	1.91 (1.13–3.25)	-
No	1.00		1.00	
Miliary TB				
Yes	0.83 (0.63–1.10)	-	1.13 (0.70–1.83)	-
No	1.00		1.00	
Any pulmonary TB-related symptoms				
Yes	0.65 (0.50–0.85)	0.70 (0.55–0.90)	0.61 (0.42–0.90)	0.46 (0.27–0.80)
No	1.00	1.00	1.00	1.00
Any systemic TB-related symptoms				
Yes	0.80 (0.69–0.93)	-	1.16 (0.60–2.26)	-
No	1.00		1.00	
Multidrug or poly-drug resistance				
Yes	0.65 (0.50–0.85)	0.44 (0.32–0.60)	0.62 (0.28–1.39)	0.28 (0.12–0.66)
No	1.00	1.00	1.00	1.00
Teaching hospital				
Yes	0.86 (0.75–0.99)	0.81 (0.71–0.92)	0.99 (0.62–1.59)	-
No	1.00	1.00	1.00	

*IQR* interquartile range, *LOS* length of stay, *HR* hazard ratio, *CI* confidence interval

^a^Models adjusted for all reported variables

^b^Cox Proportional Hazard model is modeling the time to discharge; a hazard ratio of less than one indicates a longer LOS (ie. A HR for multidrug or poly-drug resistance = 0.45 can be interpreted as: the rate of discharge from hospital is 55% slower for patients with drug resistance compared to those without drug resistance)

^c^Given strong evidence that proportional hazards assumptions did not hold for age (lines crossed in the log-log plot), we included an interaction term in the model of age (continuous, in years) multiplied by the LOS. This interaction term indicates that the effect of age was not constant throughout the length of stay

## Discussion

In a setting with universal health care access, over half of all patients were hospitalized at the time of TB diagnosis and treatment initiation. Hospitalization frequencies and LOS in our study were comparable to other US and Canadian studies [12, 14, 21]. In the one US study to differentiate initial and during-treatment hospitalizations, Taylor found that 45% of TB patients had an initial hospitalization for TB and 8% were hospitalized during treatment, with a median LOS of 11 days [14].

In Montreal Health region, there are six hospitals treating TB patients which have adequate respiratory isolation facilities. The Canadian TB Standards recommend that patients with confirmed smear-positive, culture-positive drug susceptible respiratory TB be kept under airborne precautions until there is clinical evidence of improvement, evidence of adherence to at least 2 weeks of effective treatment, and three consecutive negative sputum smears [22]. Thus some of our findings likely reflect recommended hospitalization guidelines for TB patients. However, patients may be discharged to home isolation for the period requiring airborne precautions provided there is clinical improvement, drug-resistant TB is not suspected and there is no contraindication for home isolation [22].

Our study also identified several population groups at higher hospitalization risk and longer LOS. Hospitalization risk was lowest among immigrants who arrived in Canada within 2 years of diagnosis, and increased with time since immigration, approaching rates of Canadian-born patients by 15 years post-arrival. Recent immigrant patients may have less advanced disease due to earlier diagnosis through active screening [23]. This was suggested by our study, whereby recently immigrated TB patients were less likely to have smear-positive, symptomatic pulmonary TB. These findings may also partly reflect a “healthy immigrant effect”, whereby immigrants’ health at the time of arrival is better than the Canadian-born population, an effect that tends to diminish with longer time spent in Canada [24]. Having drug resistance was associated with a higher risk of hospitalization and longer LOS—similar to the Taylor study, which found that MDR-TB cases were almost 6 times more likely to be hospitalized than non-MDR patients [14]. This has implications for resource planning, given increasing drug-resistant TB globally. There was also evidence for an increased hospitalization rate and longer LOS of homeless patients in our study, similar to previous studies [14, 25].

Strengths of this study include the virtually complete capture of confirmed TB cases in a large health region. The extraction of detailed clinical data from hospital, clinic and public health records allowed investigation into clinical and social determinants of hospitalization in the general population of TB patients. Limitations include that these data were collected retrospectively and recording of some details, such as smoking, alcohol, and illicit drug use may not be complete. Additionally, if patients were hospitalized outside Montreal, this may not have been recorded in the study database, thus underestimating hospitalization rates. It is possible that some hospitalizations were due to co-morbidities, rather than presentation of active TB symptoms (thus overestimating the TB-related hospitalization rate). These results also may not be generalizable to other jurisdictions, for example, TB hospitalizations have been shown to vary based on urban or rural residence [26].

This study adds to our knowledge about hospitalization during TB diagnosis and treatment in a large urban Canadian population. These data can be used by planners to better capture TB resource implications especially with regard to the cost effectiveness of treatment of LTBI. Our finding that homeless patients were more likely to be admitted with longer LOS, suggests a need for earlier diagnosis and treatment for these patients, and is supported by similar findings by Marks et al. in the US [25]. Our finding that Canadian-born patients tended to have more severe disease at the time of diagnosis suggests a need for interventions to reduce diagnostic delay, such as increased training for health care professionals, patients, and community members to increase their TB awareness. Some hospitalizations may also be preventable; in a US study, for example, close to 40% of hospitalizations of TB patients were considered likely to have been avoidable [1]. We found that smear-positive patients were more likely to be hospitalized with longer LOS; it is possible that in some of these cases, physicians may have been reluctant to discharge patients if they are unsure how to manage a potentially infectious patient in their home, as previously reported [1]. Professional development programs for physicians on evidence-based strategies for TB management could help to reduce time spent in-hospital. Improving community-based DOT programs could also lead to reductions in hospitalization rates of TB patients [27].

## Conclusion

We found a high hospitalization rate at the time of diagnosis and during treatment of patients with TB. Diagnostic delay due to low index of suspicion may result in patients presenting with more severe disease at the time of diagnosis. Earlier identification and treatment of patients, through interventions such as enhanced training to increase TB awareness and more targeted prevention programs, might reduce costly TB-related hospital use.

## Abbreviations

CI: Confidence interval
DOT: Directly observed therapy
HIV: Human immunodeficiency virus
HR: Hazard ratio
IQR: Interquartile range
LOS: Length of stay
LTBI: Latent tuberculosis infection
RAMQ: Régie de l’assurance maladie du Québec
SAT: Self-administered therapy
TB: Tuberculosis
WHO: World Health Organization
